# Risk factors associated with adverse events during endoscopic ultrasound-guided tissue sampling

**DOI:** 10.1371/journal.pone.0189347

**Published:** 2017-12-13

**Authors:** Kwang Hyuck Lee, Eun Young Kim, Juhee Cho, Danbee Kang, Seungmin Bang, Hyung Kil Kim, Gwang Ha Kim, Hyun Jong Choi, Joung-Ho Han, Seong Woo Jeon, Ji Kon Ryu, Jeong Seop Moon, Tae Hee Lee, Jin Woong Cho, Tae Hyeon Kim, Young Koog Cheon, Chang-Hwan Park, Jong Kyun Lee, Jong Ho Moon, Chang Min Cho

**Affiliations:** 1 Department of Medicine, Samsung Medical Center, Sungkyunkwan University School of Medicine, Seoul, Korea; 2 Department of Health Science & Technology, SAIHST, Sungkyunkwan University, Seoul, Korea; 3 Department of Medicine, Catholic University of Daegu School of Medicine, Daegu, Korea; 4 Biostatistics and Clinical Epidemiology Center, Samsung Medical Center, Sungkyunkwan University School of Medicine, Seoul, Korea; 5 Department of Health, Behavior and Society and Epidemiology, Johns Hopkins Bloomberg School of Public Health, Baltimore, United States of America; 6 Department of Medicine, Yonsei University School of Medicine, Seoul, Korea; 7 Department of Medicine, Inha University School of Medicine, Incheon, Korea; 8 Department of Internal Medicine, Pusan National University School of Medicine, Busan, Korea; 9 Department of Internal Medicine, Soonchunhyang University School of Medicine, Bucheon, Korea; 10 Department of Medicine, Chungbuk National University School of Medicine, Cheongju, Korea; 11 Department of Internal Medicine, Kyungpook National University School of Medicine, Daegu, Korea; 12 Department of Internal Medicine, Seoul National University College of Medicine, Seoul, Korea; 13 Department of Internal Medicine, Inje University College of Medicine, Seoul, Korea; 14 Institute for Digestive Research, Soonchunhyang University College of Medicine, Seoul, Korea; 15 Department of Internal Medicine, Presbyterian Medical Center, Jeonju, Korea; 16 Department of Medicine, Wonkwang University School of Medicine, Iksan, Korea; 17 Department of Medicine, Konkuk University School of Medicine, Seoul, Korea; 18 Department of Medicine, Chonnam National University School of Medicine, Gwangju, Korea; University Hospital Llandough, UNITED KINGDOM

## Abstract

**Background and aim:**

Endoscopic ultrasound-guided fine-needle aspiration (EUS-FNA) is commonly used to obtain tissue external to the gastrointestinal tract. EUS-FNA is relatively safe, but occasionally adverse events have been reported. There is scarcity of data on risk factors of adverse events. The aim of this study is to identify risk factors associated with EUS-FNA.

**Methods:**

In this multicenter case-control study, we retrospectively reviewed 4,097 cases between 2009 and 2012 at 15 hospitals in Korea. Among the patients there were 104 cases (2.5%) who had adverse events of which 12 (0.29%) were severe. We matched 520 controls (1:5 ratios) stratified by hospital to analyze the potential risk factors.

**Results:**

The most common adverse events were pancreatitis (45/104, 43.3%) and infection (46/104, 44.2%). Endoscopic retrograde cholangiopancreatography (ERCP) on the same day was a risk factor of all adverse events [OR = 2.41, 95% CI (1.41, 4.12)], pancreatitis [OR = 2.31, 95% CI (1.02, 5.25)], and infection [OR = 2.75, 95% CI (1.31, 5.78)]. More than 15 to-and-fro movements during puncture increased the risk of pancreatitis [OR = 2.30, 95% CI (1.11, 4.77)] and infection [OR = 3.65, 95% CI (1.55, 8.59)]. A higher number of punctures was positively correlated with pancreatitis [OR = 1.34, 95% CI (1.08, 1.67)] but negatively correlated with infection [OR = 0.66, 95% CI (0.48, 0.89)].

**Conclusions:**

EUS-FNA is a safe procedure in which serious adverse events are rare. We define some risk factors of adverse events during EUS-FNA, including ERCP on the same day, a higher number of punctures, and more than 15 to-and-fro movements.

## Introduction

Endoscopic ultrasound (EUS)-guided tissue sampling is a common method of pathological diagnosis for lesions around gastrointestinal tract. Frequent targets are the pancreas, peripancreatic lymph node, and sub-mucosal tumors [1–8].

To obtain a specimen, the needle must pass through gastrointestinal lumen, which can be colonized by bacteria, through connective tissue containing blood vessels, and finally reach aseptic targets. However, adverse events of this procedure are uncommon, occurring in only about 2.5% [9–13]. Adverse event rates are higher in prospective than in retrospective studies [11,14]. Adverse events occur more frequently when puncturing pancreatic cysts than solid masses. There are recommendations to avoid adverse events [9,10,15], but there have been few studies on risk factors. Most prospective studies failed to collect a sufficient number of adverse events to analyze risk factors [16–19].

To minimize EUS-guided fine-needle aspiration (EUS-FNA) adverse events, it is important to define risk factors of adverse events to help endoscopists perform safer diagnostic EUS-guided tissue sampling. We conducted a multicenter case-control study with a comprehensive search for adverse events to investigate the risk factors related to EUS-FNA.

## Methods

### Design, patients and definitions of adverse events

We undertook retrospectively a multicenter case-control study of 15 university-based hospitals in Korea. At least one investigator from each institution was a member of the EUS study group in the Korean Society of Gastrointestinal Endoscopy (Seoul, Korea). This study was approved by the Samsung Medical Center Institutional Review Board (IRB No. 2013-07-119), and the data were compiled and reported in compliance with the patient confidentiality guidelines. IRB waived the need for consent and data was accessed anonymously.

First, we reviewed all patients who underwent EUS-guided tissue diagnosis from January 2008 to December 2012 according to consensus definitions of adverse events described below. We identified 104 cases of adverse events and, classified the cases into six categories: bleeding, pancreatitis, infection, gastrointestinal perforation, seeding, and other. Bleeding was identified if hemoglobin decreased by 2 g/dL and an endoscopic or radiological study showed bleeding related to EUS-FNA or if hemoglobin decreased by 3 g/dL with no specific reason for bleeding after EUS-FNA. Pancreatitis was diagnosed in patients with a serum amylase level more than three times the upper normal limit and significant abdominal pain that started within 24 hours of the EUS-FNA and required intravenous analgesics or fasting. Gastrointestinal perforation was diagnosed in patients with abdominal pain and rigidity accompanied by intra-abdominal free air or equivalent findings with radiological examination. Infection was diagnosed in patients with a body temperature increasing from normal to no less than 38.3°C after EUS-FNA and who had no other specific cause of high temperature or who had documented infection related to EUS-FNA. Seeding along the FNA tract was identified if the tumor developed much more densely near the tract than in other locations or if it was localized along the needle tract without definite evidence of dissemination. We also defined other adverse events if they were found to be related to EUS-FNA after appropriate evaluations. We defined severe adverse events as those that required surgery, another radiological or endoscopic intervention, or admission for longer than 4 weeks. We defined categories and severity at the consensus meeting to find the clinically meaningful events objectively and consistently under this study setting.

To analyze risk factors, we selected a 1:5 ratio of matched controls stratified by hospital. Controls were assigned according to a computer-generated random number at Samsung Medical Center (Seoul, Korea). We gathered clinical data and extracted potentially modifiable details during EUS-FNA procedures. We followed 624 patients until June 2013 and the median follow up duration was 368 days. The data for 624 EUS-FNAs from all the institutions were transferred into statistical format at Samsung Medical Center. After a first-pass analysis of all data, we decided to re-collect missing data from each institution. After such two comprehensive reviews, we analyzed the final data set at Samsung Medical Center.

### Data collection for risk factors

Using a standardized clinical record form, we collected comprehensive clinical and endoscopic information from the patients’ medical records, along with endosonographic photos or videos taken during the EUS-FNA procedures This data set included demographic characteristics, experience of the endoscopist, and other procedures on the same day, the nature of the targets, and endoscopic technical aspects.

All EUS-FNA procedures were performed in inpatient settings and patients were discharged no less than 24 hours after procedures, so it should identify every case of adverse events.

### Statistical analysis

To compare baseline characteristics between cases and controls, we used a t-test for continuous variables and a Chi-square test for categorical variables. We performed univariate and multivariate analysis to find factors associated with adverse events. For the multivariate analysis, we used 2 models with increasing degrees of adjustment to account for potential confounding factors at baseline. Model 1 was adjusted for age, sex, nature of lesions, and endoscopists’ experience. Model 2 was further adjusted for variables which were statistically significant (*P* < 0.05) with univariate analysis for each outcome. To find the risk factor during EUS-FNA procedures that can be modifined by endoscopists, we also included normal pancreas puncture, distance between endoscopic tip and target, straightening endoscope during puncture, use of elevator, off-targeting method, vascular puncture, and sonographic finding of bleeding. Due to the nature of the study design, there were missing variables especially with EUS-FNA procedures, and we did analysis of these modifiable factors ony with the subjects who had information about them. All statistical analyses were performed using STATA 12.0.

## Results

### Incidence of adverse events

A total of 4,097 EUS-FNA procedures from 15 university-related hospitals. The most common indication of EUS FNA / tissue acquisition puncture sites were pathology in pancreas (86.5%) and lymph node (5.8%) (Table 1). We observed 104 (2.5%) adverse events, which included pancreatitis (*n* = 45, 43.3%), infection (*n* = 46, 44.2%), bleeding (*n* = 8, 7.7%), perforation (*n* = 1, 1%), and others (*n* = 4, 3.9%). The four unclassified adverse events were cholecystitis, pharyngeal perforation, and two cases of ileus without definite cause. There was no mortality related to procedure.

**Table 1:** Characteristics of the study population (*N* = 624).

	Cases (*n* = 104) N (%)	Controls (*n* = 520) N (%)	*P*-value
**Age (years) [Range 22–94]**	62.0 ±12.6	63.3 ±12.7	0.37
**Male**	55 (52.9)	271 (52.1)	0.89
**Alcohol drinkers**	28 (26.9)	123 (23.7)	0.48
**Smokers**	27 (26.0)	109 (21.0)	0.48
**Medical history**			
Pancreatitis	6 (5.8)	16 (3.1)	0.17
Liver cirrhosis	0	3 (0.6)	0.44
Chronic renal failure	0	5 (1.0)	0.32
Cancer	10 (9.6)	51 (9.8)	0.95
Upper GI tract surgery	2 (1.9)	42 (8.1)	0.03
**Medication**			
Antiplatelet	25 (4.8)	2 (1.9)	0.19
Anticoagulant	8 (1.5)	0	0.20
Heparinization	4 (0.8)	0	0.37
Antibiotic	198 (38.1)	42 (40.4)	0.66
Protease inhibitor	38 (7.3)	14 (13.5)	0.04
**Nature of lesion**			0.40
Solid	79 (76.0)	414 (79.6)	
Cystic	25 (24.0)	106 (20.4)	
**Malignancy**	70 (67.3)	351 (67.5)	0.97
**Origin of lesion**			0.17
Pancreas	90 (86.5)	401 (77.1)	
Lymph node	6 (5.8)	59 (11.3)	
Sub-mucosal tumor	3 (2.9)	29 (5.6)	
Other	5 (4.8)	31 (5.7)	
**Size of lesion, cm**	3.7 ±1.8	3.4 ±1.8	0.09
**Vascularity (hypovascular)**	95 (91.4)	449 (86.4)	0.22
**ERCP on the same day**	27 (26.0)	62 (11.9)	< 0.01
**Previous stent insertion**	4 (3.8)	12 (2.3)	0.37
**Experience of endoscopists (≥150)**	82 (78.9)	405 (77.9)	0.83
**Location of the endoscopic tip**			0.23
Esophagus	2 (1.9)	29 (5.6)	
Stomach	56 (53.9)	240 (45.2)	
Duodenum	44 (42.3)	229 (44.0)	
Other	1 (1.0)	3 (0.6)	
Missing	1 (1.0)	19 (3.7)	
**Size of needle**			0.04
25 G	19 (18.3)	81 (15.6)	
22 G	75 (72.1)	325 (62.5)	
19 G	7 (6.7)	82 (15.8)	
Unknown	3 (2.9)	32 (6.2)	
**Type of needle**			0.97
Conventional	89 (85.6)	439 (84.4)	
TruCut	4 (3.9)	18 (3.5)	
Procore	4 (3.9)	21 (4.0)	
Unknown	7 (6.7)	42 (8.1)	
**Number of punctures**	2.8 ±1.8	2.6 ±1.36	0.37
**Number of to-and-fro movements**			0.05
≤15	39 (37.5)	261 (50.2)	
>15	55 (52.9)	213 (41.0)	
Unknown	10 (9.6)	46 (8.8)	
**Type of adverse event**			
Pancreatitis	45 (43.3)	-	
Infection	46 (44.2)	-	
Bleeding	8 (7.7)	-	
Perforation	1 (1.0)	-	
Other	4 (3.9)	-	
**Potentially modifiable details during EUS-FNA procedures (n = 359)**^**†**^			
**Normal pancreas puncture**	37 (61.7)	141 (47.2)	0.04
**Distance between endoscope and target**	1.5± 0.9	1.3 ± 0.9	0.09
**Straightening**			0.65
Yes	39 (65.0)	205 (68.6)	
Unknown	2 (3.3)	5 (1.7)	
**Use of Elevator**			0.92
Yes	23 (38.3)	114 (38.1)	
Unknown	7 (11.7)	30 (10.0)	
**Off-targeting, yes**	2 (3.3)	3 (1.0)	0.16
**Vascular puncture, yes**	0	3 (1.0)	0.44
**Sonographic bleeding**			0.49
Yes	0	5 (1.7)	
Unknown	0	2 (0.7)	

EUS-FNA: Endoscopic ultrasound-guided fine needle aspiration

Values are mean ± SD or number (%)

^†^Patients for whom information was available about potentially modifiable details

### Risk factors of adverse events

We compared each complicated case with five matched controls stratified by hospital with regard to demographic characteristics and potentially modifiable details during EUS-FNA procedures (Tables 1 and 2). According to the institutions, clinical information, especially about technical endoscopic details such as straightening of the endoscope, puncture through visible normal pancreas, and sonographic observation of bleeding, was unavailable for more than 15% of patients. Therefore, we separately analyzed patients (*n* = 359) who had records of potentially modifiable details during EUS-FNA procedures (S1 and S2 Tables). For the multivariable analysis, we included risk factors considered to be clinically important in previous studies, such as sex, age, experience of the endoscopist, and the nature of the lesions (cystic or solid) [9,10,15]. We found several risk factors after the adjustments with model 1 and model 2 as described in statistical analysis of method section (Table 3).

**Table 2:** Factors associated with EUS-FNA adverse events (Unadjusted).

	All adverse events (*N* = 624) Odds ratio (95% CI)	Pancreatitis (*n* = 565) Odds ratio (95% CI)	Infection (*n* = 566) Odds ratio (95% CI)
**Age, years**	0.99 (0.97, 1.01)	0.98 (0.96, 1.01)	1.00 (0.97,1.03)
**Female** (reference(ref): male)	0.97 (0.64, 1.48)	0.75 (0.40, 1.40)	0.91 (0.49, 1.70)
**Drinking, yes** (ref: none)	1.19 (0.74, 1.92)	1.38 (0.70, 2.72)	1.38 (0.69, 2.76)
**Smoking, yes** (ref: none)	1.32 (0.81, 2.15)	1.09 (0.53, 2.26)	2.07 (1.05, 4.07)*
**History** (ref: none)			
Pancreatitis	1.93 (0.74, 5.05)	1.34 (0.29, 6.25)	2.66 (0.70, 10.10)
Cancer	0.98 (0.48, 2.00)	1.17 (0.43, 3.15)	0.87 (0.29, 2.57)
Surgery	0.22 (0.05, 0.94)*	0.60 (0.14, 2.60)	-
**Medication** (ref: no use)			
Medication with bleeding risk^†^	0.30 (0.07, 1.27)	0.32 (0.04, 2.48)	0.38 (0.05, 2.91)
Antibiotics	1.10 (0.72, 1.69)	3.60 (1.87, 6.94)*	0.45 (0.14, 1.45)
Protease inhibitors	1.97 (1.03, 3.79)*	2.01 (0.83, 4.83)	1.44 (0.28, 7.38)
**Nature of lesions, cyst** (ref: solid)	1.24 (0.75, 2.03)	1.58 (0.80, 3.13)	1.05 (0.48, 2.29)
**Malignancy** (ref: benign)	0.99 (0.63, 1.55)	1.07 (0.55, 2.07)	0.89 (0.46, 1.73)
**Origin of the lesion** (ref: pancreas)			
Lymph node	0.45 (0.19, 1.08)	0.15 (0.02, 1.14)	0.81 (0.27, 2.42)
Sub-mucosal tumor	0.46 (0.13, 1.54)	n.a.	0.70 (0.16, 3.11)
Other	0.72 (0.27, 1.90)	n.a.	1.22 (0.33, 4.42)
**Size of lesion, cm**	1.09 (0.98, 1.21)	0.89 (0.72, 1.10)	1.20 (1.05, 1.37)*
**Vascularity, hypovascular** (ref: hypervascular)	1.90(0.79, 4.55)	1.57 (0.46, 5.31)	2.67 (0.62, 11.50)
**ERCP on the same day, yes** (ref: none)	2.59 (1.56, 4.32)*	2.82 (1.31, 6.10)*	2.73 (1.35, 5.53)*
**Previous stent insertion, yes** (ref: none)	1.69 (0.53, 5.36)	2.79 (0.72, 10.79)	1.52 (0.17, 13.17)
**Experience of endoscopists, ≥150** (ref: < 150)	1.06 (0.63, 1.77)	0.83 (0.40, 1.71)	0.64 (0.20, 2.02)
**Location of the endoscopic tip** (ref: esophagus)			
Stomach	3.38 (0.78, 14.60)	n.a.	0.82 (0.27, 2.42)
Duodenum	2.79 (0.64, 12.10)	n.a.	0.70 (0.16, 3.11)
Etc.	4.83 (0.33, 70.40)	n.a.	1.22 (0.34, 4.42)
**Size of needle** (ref: 25 G)			
22 G	0.98 (0.56, 1.72)	0.61 (0.29, 1.28)	1.18 (0.30, 4.74)
19 G	0.36 (0.15, 0.91)*	0.22 (0.05, 1.10)	0.38 (0.07, 2.15)
Unknown	0.40 (0.11, 1.44)	0.17 (0.02, 1.39)	1.33 (0.20, 8.86)
**Type of needle** (ref: conventional)			
TruCut	1.10 (0.36, 3.32)	n.a.	0.66 (0.08, 5.34)
Procore	0.94 (0.31, 2.80)	n.a.	1.16 (0.12, 10.91)
Unknown	0.82 (0.36, 1.89)	n.a.	1.63 (0.35, 7.74)
**Number of punctures**	1.07 (0.92, 1.23)	1.24 (1.02, 1.50)*	0.70 (0.51, 0.96)*
**To-and-fro movement** (ref:1–15)			
>15	1.73 (1.10, 2.71)*	2.25 (1.07, 4.73)*	2.74 (1.13, 6.61)*
Unknown	1.45 (0.68, 3.12)	1.51 (0.45, 5.08)	1.60 (0.38, 6.65)

EUS-FNA: Endoscopic ultrasound-guided fine needle aspiration; ERCP: endoscopic retrograde cholangiopancreatography; n.a.: not available

^†^Antiplatelet drugs, anticoagulants, or heparinization.

*P < 0.05

**Table 3:** Factors associated with EUS-FNA adverse events (Adjusted).

	All adverse events (*n* = 624)		Pancreatitis (*n* = 565)		Infection (*n* = 566)	
	Model 1^†^ Odds ratio (95% CI)	Model 2^†^ Odds ratio (95% CI)	Model 1^†^ Odds ratio (95% CI)	Model 2^‡^ Odds ratio (95% CI)	Model 1^†^ Odds ratio (95% CI)	Model 2^‡^ Odds ratio (95% CI)
**ERCP on the same day** (ref: none)	2.58 (1.54, 4.32)*	2.41 (1.41, 4.12)*	2.36 (1.04, 5.32)*	2.31 (1.02, 5.25)*	2.77 (1.36, 5.66)*	2.75 (1.31, 5.78)*
**To-and-fro movements** (ref:1–15)				-		
>15	1.87 (1.17, 3.01)*	1.57 (0.95, 2.60)	2.87 (1.30, 6.32)*	2.30 (1.11, 4.77)*	2.93 (1.17, 7.30)*	3.65 (1.55, 8.59)*
Unknown	1.43 (0.65, 3.12)	1.76 (0.76, 4.04)	1.48 (0.43, 5.13)	1.24 (0.36, 4.31)	1.43 (0.33, 6.35)	2.13 (0.53, 8.66)
**Size of needle** (ref: 25 G)			-	-	-	-
22 G	0.95 (0.54, 1.67)	0.88 (0.48, 1.59)	-	-	-	-
19 G	0.32 (0.12, 0.81)*	0.35 (0.13, 0.92)*	-	-	-	-
Unknown	0.36 (0.10, 1.32)	0.36 (0.09, 1.40)				
**History of surgery** (ref: none)	0.23 (0.05, 0.96)*	0.20 (0.05, 0.84)*				
**Protease inhibitor** (ref: no use)	2.03 (1.03, 3.99)*	1.95 (0.94, 4.05)				
**Number of punctures**	-	-	1.33 (1.08, 1.63)*	1.34 (1.08, 1.67)*	0.69 (0.50, 0.96)*	0.66 (0.48, 0.89)*
**Antibiotics** (ref: no use)	-	-	3.36 (1.69, 6.69)*	3.78 (1.82, 7.84)*	-	-
**Smoking, yes** (ref: none)	-	-	-	-	2.25 (1.06, 4.78)*	2.60 (1.17, 5.77)*
**Size of lesion, cm**	-	-	-	-	1.21 (1.05, 1.40)	1.18 (1.01, 1.36)*

EUS-FNA: Endoscopic ultrasound-guided fine needle aspiration; ERCP: Endoscopic retrograde cholangiopancreatography; ref: Reference

^†^ Model 1 adjusted for age, sex, nature of lesion, and experience of endoscopists

^‡^ Model 2 additionally adjusted for variables that were statistically significant (*P* < 0.05) in the univariate analysis (Table 2) All adverse events: ERCP on the same day, to-and-fro movements >15, size of needle, history of surgery, and protease inhibitor; Pancreatitis: ERCP on the same day, to-and-fro movements >15, number of punctures, and antibiotics; Infection: ERCP on the same day, to-and-fro movements >15, number of punctures, smoking and size of lesion.

*P < 0.05

There were some consistent factors which increased the risk of all adverse events, pancreatitis and infections. The risk of all adverse events, pancreatitis and infection was higher in patients with ERCP on the same day. More than 15 to-and-fro movements of the needle carried higher risk in all adverse events, pancreatitis and infection except model 2 of all adverse events. A higher number of punctures was related with higher risk of pancreatitis but lower risk of infection. Therefore, the risk of all adverse events was not changed with the number of punctures. Size of needle, history of surgery, use of protease inhibitor, use of antibiotics, smoking and the size of lesion were related with the risk but they were not consistent in all adverse events and models (Table 3). The most common puncture site was pancreas, so we analyzed the risk factors of all adverse events and pancreatitis in 495 patients who experienced pancreas puncture during EUS-FNA. ERCP on the same day and more than 15 to-and-fro movements correlated with all adverse events and pancreatitis (Table 4).

**Table 4:** Risk factors among patients with EUS-FNA to the pancreas^†^ (Adjusted).

	All adverse events (*n* = 491)		Pancreatitis (*n* = 445)	
	Model 1^‡^ OR (95% CI)	Model 2^§^ OR (95% CI)	Model 1^‡^ OR (95% CI)	Model 2^§^ OR (95% CI)
**Bile duct abnormal** (ref: none)	2.35 (1.29, 4.27)*	1.89 (0.98, 3.67)		
**ERCP on the same day** (ref: none)	2.42 (1.40, 4.17)*	1.90 (1.04, 3.46)*	2.56 (1.15, 5.68)*	2.15 (0.93, 4.98)
**To-and-fro movements** (ref:1–15)				
>15	2.13 (1.27, 3.57)*	2.12 (1.25, 3.59)*	2.67 (1.19, 5.96)*	2.47 (1.09, 5.59)*
Unknown	1.63 (0.67, 3.97)	1.74 (0.70, 4.29)	1.70 (0.48, 6.05)	1.43 (0.33, 6.16)
**Number of punctures**			1.30 (1.05, 1.62)*	1.22 (0.97, 1.53)

^†^All adverse events (*n* = 90); pancreatitis (*n* = 44)

EUS-FNA: Endoscopic ultrasound-guided fine needle aspiration; ERCP: Endoscopic retrograde cholangiopancreatography; ref: Reference

^‡^ Model 1 adjusted for age, sex, nature of lesion, and experience of endoscopists

^§^ Model 2 additionally adjusted for variables that were statistically significant (P < 0.05) in the univariate analysis (S2); all adverse events: ERCP on the same day, to-and-fro movements >15, and bile duct abnormal; pancreatitis: ERCP on the same day, to-and-fro movements >15, and number of punctures

*P < 0.05

Analysis of patients with sufficient information about potentially modifiable details during EUS-FNA procedures showed that ERCP on the same day [OR = 2.53, 95% CI (1.21, 5.32)] and normal pancreas puncture [OR = 1.89 CI (1.03, 3.47)] correlated with all adverse events (S2 Table). Risk factors of pancreatitis in 282 patients who experienced pancreas puncture during EUS-FNA were ERCP on the same day [OR = 3.07 CI (1.16, 8.12)], more than 15 to-and-fro movements [OR = 3.58 CI (1.45, 8.86)], and normal pancreas puncture [OR = 2.78 CI (1.14, 6.80)] (S3 and S4 Tables).

### Incidence of severe adverse events

Severe adverse events occurred in 0.29% (12/4097) of all procedures. Of 45 cases with pancreatitis, 3 had severe events: One received an endoscopic cystogastrostomy 4 weeks after EUS-FNA, and the other two patients were hospitalized for longer than 4 weeks. Four patients from 46 infection cases required drainage to control. Two infected cysts were drained internally with plastic stents and another infected cyst was drained externally with a nasocystic tube for irrigation. Fourth patient who underwent ERCP on the same day needed ERBD revision. Two of eight bleeding cases were severe: one needed endoscopic coagulation and the other underwent angiography and surgical correction. During duodenal intubation one patient underwent a gastrointestinal perforation that required a surgery for primary closure. Two severe adverse events from four patients classified as others were an acute cholecystitis and a pharyngeal perforation. The acute cholecystitis developed during EUS-FNA for gallbladder cancer and managed with percutaneous transhepatic gallbladder drainage. The pharyngeal perforation during oral endoscopic intubation was managed with initial incision and drainage and delayed surgical repair.

All adverse event cases recovered without mortality.

## Discussion

The incidence of adverse events in EUS-FNA was quite low, as expected, in this comprehensive nationwide retrospective review of medical records in Korea, where EUS-FNA has become popular. Infection and pancreatitis were most common. Fortunately, clinically severe adverse events occurred in 0.29% of all cases without mortality. The severe adverse event rate of the survey in Japan was 0.23%, which is consistent with our results [20].

The overall complication rate in this study was 2.5% that seemed to be higher than that of previous retrospective studies [11,14,21]. In our practice, the patients were observed in admission at least for 24 hours after EUS-FNA and we comprehensively collected the data. So, the complication rate was similar to that of prospective studies [9,10,12,13,21].

This multicenter case-control study collected sufficient events to analyze risk factors, including endoscopic techniques after comprehensive reviews of the largest number of EUS-FNA procedures in a single study to date. The major risk factors we found were ERCP on the same day, a higher number of punctures, and increasing to-and-fro movements during puncture.

In this study, ERCP following EUS FNA on the same day was associated with higher risk of adverse events than EUS-FNA alone. Because EUS-FNA and ERCP were usually done together in practice, it is necessary to assess the risk of ERCP and EUS-FNA simultaneously. However, no prospective study has assessed the risk of a combined procedure in the same day. There are only two retrospective observational studies with small number of patients; one study in 2007 reported that a combined procedure might be safe with only 25 patients [22] and the other in 2012 that the complication rate of stent and EUS-FNA seemed not to be higher than that of stent alone [23]. In the later study, the stent alone group consisted of patients with higher risk of adverse events (higher American Society of Anesthesiologists score [p = 0.011] and older age [p = 0.06]). As the ERCP procedures itself carry a high risk of post-procedural adverse events, further study should be necessary to evaluate the effect of combined procedure on adverse events.

Higher number of punctures increased the risk of pancreatitis and decreased that of infection, so it did seem not to correlate with the overall adverse event rate. The puzzling result that a larger number of punctures was inversely related to the development of infection might stem from selection bias by indication. In cases with a high risk of infection in the clinical context, an endoscopist tries to minimize pancreatic puncture numbers, especially into a pancreatic cyst. Therefore, we found fewer punctures of cysts than of solid masses in this study: 2.9 passes in solid masses and 1.9 passes in cysts (*P* < 0.001). Even though cysts have been reported to be more vulnerable to infection than solid masses [24–26], cyst punctures did not increase risk of infection in our study, apparently because most institutes followed the rules of cyst aspiration: complete aspiration if possible and prophylactic antibiotics. Because of those routine protocols, we found no difference according to the nature of lesions. Similarly, the incidence of infection during cystic punctures in prospective studies using prophylactic antibiotics was low [16,19,27].

More than 15 to-and-fro movements was a risk factor in pancreatitis and infections but not in overall adverse events. The other adverse events, except pancreatitis and infections, included bleeding and duodenal and pharyngeal perforation during intubation. Those adverse events prevented the endosonographers from performing a higher number of to-and-fro movements; therefore, we observed more than 15 to-and-fro movements in 15% of those patients, compared with 44% and 74% of patients with pancreatitis and infections, respectively (*P* = 0.004). Therefore, more movement was not a risk factor of all adverse events in this study.

These three factors, ERCP on the same day, number of punctures, and number of to-and-fro movements, might be considered in patients for whom the development of a mild adverse event might result in a serious problem because of the clinical situation. In such high-risk patients, ERCP and EUS might be performed on different days, and the endoscopist could try to limit the numbers of to-and-fro movements and punctures.

We also identified other factors related to overall or specific adverse events: 19G needle use, history of upper gastrointestinal tract surgery, use of antibiotics, smoking, larger lesions, and passage through normal pancreas during lesion puncture. Some of those results might have been caused by bias or confounded by indication. Usually, endosonographers avoid stiff 19-gauge needles in lesions that are more difficult to access and are cautious and less aggressive with patients who have abnormal anatomy. Therefore, use of a 19G needle and a history of surgery negatively correlated with adverse events. Moreover, there were only 12 EUS-FNA done to patients with the altered anatomy among 44 patients who underwent surgeries. This could be another reason of negative correlation between a history of upper abdominal surgery and adverse events after EUS-FNA. Clinicians are more likely to administer antibiotics to patients with a higher risk of adverse events, so use of antibiotics is positively related to development of adverse events.

We analyzed the size as a continuous variable and it didn’t relate with any adverse events. Katanuma A et al reported that the incidence of adverse events after EUS-FNA was significantly increased in small tumors (≤20 mm) [28] but there was no statistical difference when we assess whether tumor 20 mm or less were a risk factor. (Control: Case = 35.9%: 26.5% p = 0.07)

Another limitation of this study was that there were considerably missing variables, which could bias the actual risk. To overcome it, we analyzed the data after excluding patients with missing variables to assess whether those variables were risk factors. In that analysis, we found new risk factors consistent with that of all patients. One of them was passage through the normal pancreas to puncture the lesion which increased the risk like a previous report [15]. And, pancreatic trauma is a well-known etiology of pancreatitis.

From this study, we found some risk factors might be associated with complication of EUS-FNA. The results of our study would be helpful for endoscopists to perform safer EUS-FNA. Further prospective study would be necessary to determine risk factors of EUS-FNA.

## Supporting information

S1 Table Factors associated with adverse events among patients with information about potentially modifiable details during EUS-FNA procedures (Unadjusted). (DOCX)

S2 Table Factors associated with adverse events among patients with information about potentially modifiable details during EUS-FNA procedures (Adjusted). (DOCX)

S3 Table Factors associated with pancreatitis among patients with information about potentially modifiable details during EUS-FNA procedures (Adjusted). (DOCX)

S4 Table Factors associated with adverse events† of EUS-FNA to the pancreas (Unadjusted). (DOCX)

S1 Data Dataset and coding file: It consists of the raw data set without patient identifications and explanations of each variable. (XLSX)
