# Supplementary material for: Risk factors associated with adverse events during endoscopic ultrasound-guided tissue sampling
Source: PLoS One. 2017 Dec 13;12(12):e0189347. doi: 10.1371/journal.pone.0189347 (PMC5728556; doi:10.1371/journal.pone.0189347)
Supplement: S1 Table — (DOCX) [file pone.0189347.s001.docx]

**S1 Table. Factors associated with adverse events among patients with information about potentially modifiable details during EUS-FNA procedures (Unadjusted)**

|  | **All regions^†^** | |  | **To the pancreas^‡^** | |
| --- | --- | --- | --- | --- | --- |
|  | **All adverse events (*n* = 359) Odds ratio (95 % CI)** | **Pancreatitis (*n* = 336) Odds ratio (95 % CI)** |  | **All adverse events (*n* = 282) Odds ratio (95 % CI)** | **Pancreatitis (*n* = 268) Odds ratio (95 % CI)** |
| **Age, years** | 0.99 (0.97, 1.01) | 0.98 (0.96, 1.01) |  | 0.98 (0.96, 1.01) | 0.98 (0.95, 1.01) |
| **Female** (reference(ref): male) | 0.95 (0.55, 1.66) | 0.83 (0.42, 1.66) |  | 0.98 (0.53, 1.82) | 0.86 (0.42, 1.74) |
| **Drinking, yes** (ref: none) | 0.98 (0.52, 1.85) | 1.09 (0.50, 2.35) |  | 1.01 (0.50, 2.02) | 0.96 (0.43, 2.15) |
| **Smoking, yes** (ref: none) | 0.69 (0.34, 1.41) | 0.85 (0.38, 1.95) |  | 0.91 (0.43, 1.95) | 1.04 (0.44, 2.42) |
| **History** (ref: none) |  |  |  |  |  |
| Pancreatitis | 1.87 (0.57, 6.08) | 1.49 (0.32, 7.03) |  | 1.93 (0.58, 6.42) | 1.31 (0.27, 6.22) |
| Cancer | 1.39 (0.57, 3.56) | 1.27 (0.42, 3.87) |  | 1.58 (0.41, 6.06) | 2.25 (0.58, 8.75) |
| Surgery | 0.51 (0.12, 2.24) | 0.84 (0.19, 3.77) |  | 0.83 (0.18, 3.90) | 1.18 (0.25, 5.56) |
| **Medication** (ref: no use) |  |  |  |  |  |
| Medication with bleeding risk^a^ | 0.24 (0.03, 1.79) | 0.46 (0.06, 3.37) |  | 0.32 (0.04, 2.47) | 0.44 (0.07, 3.49) |
| Antibiotics | 1.05 (0.59, 1.88) | 2.05 (0.91, 4.64) |  | 0.98 (0.52, 1.87) | 1.77 (0.77, 4.07) |
| Protease inhibitors | 2.09 (0.97, 4.34) | 1.89 (0.81, 4.45) |  | 2.05 (0.97, 4.34) | 1.85 (0.77, 4.43) |
| **Nature of lesions, cyst** (ref: solid) | 1.18 (0.63, 2.34) | 1.57 (0.75, 3.28) |  | 1.21 (0.63, 2.34) | 1.28 (0.61, 2.72) |
| **Malignancy** (ref: benign) | 1.20 (0.67, 2.15) | 1.11 (0.54, 2.26) |  | 1.08 (0.57, 2.06) | 0.99 (0.47, 2.05) |
| **Origin of the lesion** (ref: pancreas) |  |  |  |  |  |
| Lymph node | 0.53 (0.18, 1.56) | 0.18 (0.02, 1.39) |  | - | - |
| Sub-mucosal tumor | 0.58 (0.13, 2.60) | n.a. |  | - | - |
| Other | 1.16 (0.37, 3.62) | n.a. |  | - | - |
| **Size of lesion, cm** | 1.07 (0.92, 1.24) | 0.89 (0.70, 1.13) |  | 1.05 (0.90, 1.25) | 0.90 (0.71, 1.14) |
| **Vascularity, hypovascular** (ref: hypervascular) | 1.59 (0.53, 4.70) | 1.96 (0.45, 8.61) |  | 2.43 (0.55, 10.73) | 1.70 (0.38, 7.62) |
| **Surrounding structure** (ref: normal) |  |  |  |  |  |
| Pancreatic duct: abnormal | - | - |  | 1.10 (0.56, 2.15) | 1.28 (0.61, 2.72) |
| Bile duct: abnormal | - | - |  | 1.28 (0.60, 2.70) | 1.09 (0.44, 2.66) |
| **ERCP on the same day, yes** (ref: none) | 3.10 (1.54, 6.24)* | 3.93 (1.77, 8.79)* |  | 3.08 (1.47, 6.46) | 3.48 (1.53, 7.90)* |
| **Previous stent insertion, yes** (ref: none) | 1.69 (0.45, 6.45) | 1.84 (0.38, 8.87) |  | 1.78 (0.46, 6.99) | 1.65 (0.34, 8.08) |
| **Experience of endoscopists, ≥150** (ref: < 150) | 1.13 (0.63, 2.03) | 1.18 (0.57, 2.44) |  | 1.16 (0.61, 2.23) | 1.24 (0.58, 2.65) |
| **Location of the endoscopic tip** (ref: esophagus) |  |  |  |  |  |
| Stomach | 3.72 (0.48, 28.98) | n.a. |  | n.a. | n.a. |
| Duodenum | 4.13 (0.53, 32.26) | n.a. |  | n.a. | n.a. |
| Etc. | 18.00 (0.58, 553.59) | n.a. |  | n.a. | n.a. |
| **Size of needle** (ref: 25 G) |  |  |  |  |  |
| 22 G | 0.86 (0.46, 1.62) | 0.63 (0.30, 1.32) |  | 1.04 (0.52, 2.08) | 0.83 (0.39, 1.78) |
| 19 G | 0.64 (0.20, 2.01) | 0.41 (0.09, 1.94) |  | 1.05 (0.31, 3.58) | 0.61 (0.12, 2.94) |
| Unknown | 0.43 (0.12, 1.57) | 0.18 (0.02, 1.47) |  | 0.37 (0.08, 1.75) | 0.21 (0.03, 1.73) |
| **Type of needle** (ref: conventional) |  |  |  |  |  |
| TruCut | 1.23 (0.33, 4.55) | 1.72 (0.46, 6.42) |  | 2.69 (0.62, 11.72) | 3.44 (0.79, 15.15) |
| Procore | 1.10 (0.35, 3.39) | n.a. |  | 1.00 (0.21, 4.79) | n.a. |
| Unknown | 0.82 (0.35, 1.94) | 0.16 (0.02, 1.23) |  | 0.53 (0.18, 1.57) | 0.17 (0.02, 1.28) |
| **Number of punctures** | 1.20 (1.01, 1.44)* | 1.25 (1.03, 1.55)* |  | 1.18 (0.98, 1.43) | 1.24 (1.01, 1.53)* |
| **To-and-fro movement** (ref:1–15) |  |  |  |  |  |
| >15 | 1.75 (0.91, 3.36) | 3.00 (1.41, 6.36)* |  | 2.00 (1.00, 3.96) | 3.00 (1.39, 6.50)* |
| Unknown | 1.77 (0.80, 3.90) | 1.71 (0.59, 4.94) |  | 2.24 (0.90, 5.55) | 2.27 (0.76, 6.81) |
| **Technical aspects** |  |  |  |  |  |
| Normal pancreas puncture, yes | 1.80 (1.02, 3.18)* | 3.12 (1.40, 6.95)* |  | 1.84 (1.00, 3.46)* | 2.47 (1.16, 5.27)* |
| Distance between endoscope and target, cm | 1.29 (0.96, 1.74) | 1.33 (0.67, 2.64) |  | 1.29 (0.96, 1.74) | 1.15 (0.75, 1.77) |
| Straightening, yes | 0.89 (0.49, 1.63) | 0.61 (0.30, 1.23) |  | 0.95 (0.49. 1.84) | 0.95 (0.49. 1.84) |
| Use of elevator, yes | 1.04 (0.58, 1.89) | 1.44 (0.71, 2.92) |  | 0.91 (0.48, 1.73) | 0.91 (0.48, 1.73) |
| Off-targeting, yes | 3.40 (0.56, 20.81) | 5.63 (0.91, 34.91) |  | 3.18 (0.51, 19.55) | 3.18 (0.52, 19.55) |
| Vascular puncture, yes | 0.70 (0.15, 3.17) | 1.06 (0.23, 4.87) |  | 0.69 (0.15, 3.15) | 0.69 (0.15, 3.17) |

^†^ All adverse events (*n* = 60); pancreatitis (*n* = 37)

^‡^ All adverse events (*n* = 50); pancreatitis (*n* = 36)

EUS-FNA: Endoscopic ultrasound-guided fine needle aspiration; ERCP: Endoscopic retrograde cholangiopancreatography; ; n.a.: Not available

**P* < 0.05
