# Supplementary material for: Risk factors associated with adverse events during endoscopic ultrasound-guided tissue sampling
Source: PLoS One. 2017 Dec 13;12(12):e0189347. doi: 10.1371/journal.pone.0189347 (PMC5728556; doi:10.1371/journal.pone.0189347)
Supplement: S2 Table — (DOCX) [file pone.0189347.s002.docx]

**S2 Table. Factors associated with adverse events among patients with information about potentially modifiable details during EUS-FNA procedures (Adjusted)**

|  | **All regions (*n* = 359)^†^** | | **To the pancreas (*n* = 282)^†^** | |
| --- | --- | --- | --- | --- |
|  | **Model 1^‡^ OR (95 % CI)** | **Model 2^§^ OR (95 % CI)** | **Model 1^‡^ OR (95 % CI)** | **Model 2^§^ OR (95 % CI)** |
| **ERCP on the same day** (ref: none) | 3.21 (1.58, 6.50)* | 2.53 (1.21, 5.32)* | 3.36 (1.57, 7.18)* | 3.07 (1.42, 6.64)* |
| **Number of punctures** | 1.24 (1.02, 1.51)* | 1.19 (0.97, 1.46) |  |  |
| **Normal pancreas puncture, yes** | 1.94 (1.08, 3.46)* | 1.89 (1.03, 3.47)* | 1.97 (1.03, 3.77)* | 1.76 (0.91, 3.41) |

^†^All adverse events among patients with EUS-FNA to any region (*n* = 60); pancreatitis among patients with EUS-FNA to the pancreas (*n* = 50)

EUS-FNA: Endoscopic ultrasound-guided fine needle aspiration; ERCP: Endoscopic retrograde cholangiopancreatography; ref: Reference

^‡^ Model 1 adjusted for age, sex, nature of lesion, and experience of endoscopists

^§^ Model 2 additionally adjusted for ERCP on the same day, number of punctures, and normal pancreas puncture

**P* < 0.05
