# Supplementary material for: Risk factors associated with adverse events during endoscopic ultrasound-guided tissue sampling
Source: PLoS One. 2017 Dec 13;12(12):e0189347. doi: 10.1371/journal.pone.0189347 (PMC5728556; doi:10.1371/journal.pone.0189347)
Supplement: S3 Table — (DOCX) [file pone.0189347.s003.docx]

**S3 Table. Factors associated with pancreatitis among patients with information about potentially modifiable details during EUS-FNA procedures (Adjusted)**

|  | **All regions (*n* = 336)^†^** | | **To the pancreas (*n* = 268)^†^** | |
| --- | --- | --- | --- | --- |
|  | **Model 1^‡^**  **OR (95 % CI)** | **Model 2^§^**  **OR (95 % CI)** | **Model 1^‡^**  **OR (95 % CI)** | **Model 2^§^**  **OR (95 % CI)** |
| **ERCP on the same day** (ref: none) | 4.25 (1.86, 9.72)* | 2.98 (1.20, 7.40)* | 3.81 (1.63, 8.90)* | 2.83 (1.12, 7.20)* |
| **Number of punctures** | 1.35 (1.08, 1.69)* | 1.21 (0.96, 1.53) | 1.31 (1.04, 1.64)* | 1.16 (0.92, 1.49) |
| **Normal pancreas puncture, yes** | 3.45 (1.53, 7.77)* | 2.53 (1.15, 5.58)* | 2.81 (2.81, 6.14)* | 2.59 (1.14, 5.92)* |
| **To-and-fro movements** (ref:1–15) |  |  |  |  |
| >15 | 3.86 (1.72, 8.67)* | 3.40 (1.46, 7.88)* | 3.49 (1.52, 7.98)* | 3.14 (1.32, 7.52)* |
| Unknown | 1.97 (0.64, 6.05) | 1.91 (0.52, 7.01) | 2.46 (0.76, 7.90) | 2.21 (0.58, 8.44) |

^†^Pancreatitis among patients with EUS-FNA to any region (n=37); pancreatitis among patients with EUS-FNA to the pancreas (*n* = 36)

EUS-FNA: Endoscopic ultrasound-guided fine needle aspiration; ERCP: Endoscopic retrograde cholangiopancreatography; ref: Reference

^‡^ Model 1 adjusted for age, sex, nature of lesion, and experience of endoscopists

^§^ Model 2 additionally adjusted for ERCP on the same day, number of punctures, normal pancreas puncture, and to-and-fro movements >15

**P* < 0.05
