# Supplementary material for: Risk factors associated with adverse events during endoscopic ultrasound-guided tissue sampling
Source: PLoS One. 2017 Dec 13;12(12):e0189347. doi: 10.1371/journal.pone.0189347 (PMC5728556; doi:10.1371/journal.pone.0189347)
Supplement: S4 Table — (DOCX) [file pone.0189347.s004.docx]

**S4 Table. Factors associated with adverse events^†^ of EUS-FNA to the pancreas (Unadjusted)**

|  | **All adverse events (*n* = 491)**  **Odds ratio (95 % CI)** | **Pancreatitis (*n* = 445)**  **Odds ratio (95 % CI)** |
| --- | --- | --- |
|  |  |  |
| **Age, year** | 0.99 (0.97, 1.01) | 0.97 (0.96, 1.00) |
| **Female** (reference (ref): male) | 0.96 (0.60, 1.51) | 0.76 (0.40, 1.44) |
| **Drinking, yes** (ref: none) | 1.29 (0.77, 2.15) | 1.27 (0.63, 2.57) |
| **Smoking, yes** (ref: none) | 1.83 (1.08, 3.08)* | 1.37 (0.65, 2.89) |
| **History** (ref: none) |  |  |
| Pancreatitis | 1.97 (0.73, 5.29) | 1.23 (0.26, 5.80) |
| Cancer | 1.06 (0.39, 2.90) | 2.11 (0.66, 6.74) |
| Surgery | 0.39 (0.09, 1.70) | 0.87 (0.19, 3.91) |
| **Medication** (ref: no use) |  |  |
| Antiplatelet | 0.10 (0.09, 2.00) | 0.43 (0.05, 3.43) |
| **Prophylaxis** (ref: no use) |  |  |
| Antibiotics | 0.99 (0.62 1.58) | 3.33 (1.71, 6.48)* |
| Protease inhibitors | 1.84 (0.90, 3.73) | 1.96 (0.80, 4.78) |
| **Nature of lesions,** **cyst** (ref: solid) | 1.11 (0.66, 1.86) | 1.30 (0.65, 2.59) |
| **Malignant or benign, malignant** (ref: benign) | 0.88 (0.54, 1.44) | 0.96 (0.49, 1.89) |
| **Size of lesion, cm** | 1.09 (0.97, 1.22) | 0.89 (0.71, 1.10) |
| **Vascularity,** **hypovascular** (ref: hypervascular) | 3.23 (0.97, 10.68) | 1.45 (0.42, 5.02) |
| **Surrounding structure** (ref: normal) |  |  |
| Pancreatic duct: abnormal | 1.41 (0.81, 2.46) | 1.47 (0.70, 3.06) |
| Bile duct: abnormal | 2.14 (1.21, 3.78)* | 1.28 (0.55, 2.99) |
| **ERCP on the same day** (ref: none) | 2.42 (1.41, 4.16)* | 2.54 (1.16, 5.56) |
| **Previous stent insertion** (ref: none) | 1.82 (0.56, 5.93) | 2.70 (0.67, 10.83) |
| **Endoscopist experience,** **≥150** (ref: < 150) | 1.04 (0.59, 1.84) | 0.86 (0.39, 1.85) |
| **Size of needle** (ref: 25G) |  |  |
| 22G | 1.21 (0.66, 2.23) | 0.79 (0.37, 1.68) |
| 19G | 0.50 (0.19, 1.29) | 0.28 (0.06, 1.40) |
| Unknown | 0.36 (0.08, 1.68) | 0.20 (0.03, 1.63) |
| **Type of needle** (ref: conventional) |  |  |
| TruCut | 2.50 (0.71, 9.74) | n.a. |
| Procore | 0.87 (0.19, 4.07) | n.a. |
| Unknown | 0.51 (0.18, 1.49) | n.a. |
| **Number of punctures** | 1.06 (0.91, 1.23) | 1.24 (1.01, 1.51)* |
| **To-and-fro movements** (ref:1–15) |  |  |
| >15 | 1.97 (1.20, 3.21)* | 1.33 (1.08, 5.01)* |
| Unknown | 1.73 (0.73, 4.13) | 1.83 (0.53, 6.35) |

^†^ All adverse events (*n* = 90); pancreatitis (*n* = 44)

EUS-FNA: Endoscopic ultrasound-guided fine needle aspiration; ERCP: Endoscopic retrograde cholangiopancreatography; n.a.: Not available

**P* < 0.05
